# Stage-Specific Serum Proteomic Signatures Reveal Early Biomarkers and Molecular Pathways in Huntington’s Disease Progression

**DOI:** 10.3390/cells14151195

**Published:** 2025-08-04

**Authors:** Christiana C. Christodoulou, Christiana A. Demetriou, Eleni Zamba-Papanicolaou

**Affiliations:** 1 Neuroepidemiology Department, The Cyprus Institute of Neurology and Genetics, Nicosia 2371, Cyprus; 2 Department of Primary Care and Population Health, University of Nicosia Medical School, Nicosia 2371, Cyprus; demetriou.chri@unic.ac.cy

**Keywords:** Huntington’s Disease, proteomics, serum analysis, proteins, bioinformatics, pathway analysis, protein-protein interaction networks, biomarkers

## Abstract

Background: Huntington’s Disease (HD) is a monogenic neurodegenerative disease resulting in a CAG repeat expansion in the *HTT* gene. Despite this genetic simplicity, its molecular mechanisms remain highly complex. Methods: In this study, untargeted serum proteomics, bioinformatics analysis, biomarker filtering and ELISA validation were implemented to characterize the proteomic landscape across the three HD stages—asymptomatic, early symptomatic and symptomatic advanced—alongside gender/age-matched controls. Results: We identified 84 over-expressed and 118 under-expressed differentially expressed proteins. Enrichment analysis revealed dysregulation in pathways including the complement cascade, LXR/RXR activation and RHOGDI signaling. Biomarker analysis highlighted key proteins with diagnostic potential, including CAP1 (*AUC* = 0.809), CAPZB (*AUC* = 0.861), TAGLN2 (*AUC* = 0.886), THBS1 (*AUC* = 0.883) and CFH (*AUC* = 0.948). CAP1 and CAPZB demonstrated robust diagnostic potential in linear mixed-effects models. CAP1 decreased in the asymptomatic stage, suggesting early cytoskeletal disruption, while CAPZB was consistently increased across HD stages. Conclusions: Our findings illuminate the dynamic proteomic and molecular landscape of HD. Future studies should validate these candidates in larger, more diverse cohorts and explore their mechanistic roles in HD pathology and progression.

## 1. Introduction

Huntington’s Disease (HD) is a rare, autosomal dominant inherited neurodegenerative disease with a severely progressive phenotype, primarily affecting the medium spiny neurons (MSNs) of the basal ganglia, with a pivotal role in motor control, executive function and behavior [1,2]. HD results from a cytosine–adenine–guanine (CAG) trinucleotide repeat expansion in the *Huntingtin* (HTT) gene, encoding the HTT protein, located on Chromosome 4, exon 1 [1]. Mutant HTT (mHTT) disrupts various cellular processes, including transcription, intracellular transport and synaptic function [1]. The number of CAG repeat expansions is a key predictor of age of onset (AOO) and disease severity [1]. In healthy individuals, the CAG trinucleotide is normally repeated between 10 and 35 times [1]. Individuals having between 36 and 39 CAG repeats may or may not develop HD, meaning there is reduced penetrance [1]. However, individuals with 40 or more CAG repeats will always develop signs and symptoms of the disease [1]. The clinical phenotype includes movement impairment (involuntary choreic movement), behavioral impairment (depression) and cognitive impairment (lapses in short-term memory) [2]. The typical AOO is 40 years, with an average life expectancy of 17 years after symptom onset [2]. In Cyprus, the prevalence of HD was 4.64 per 10,000 population (95% CI: 3.30–6.34), and the incidence 0.12 per 100,000 population (95% CI: 0.00–0.66) in a 2015 study [3]. 

Proteomics is the comprehensive study of all proteins: their interactions, functions, composition, structure and cellular activities within a biological system under different conditions and disease stages [4]. The proteome is the complete set of proteins expressed within the biological sample of interest [4]. Proteomics (i) reveals critical protein(s) with key functions in disease etiology, (ii) identifies proteins involved in disease pathology and (iii) drives functional interpretation by identifying modified proteins in dysregulated cellular/molecular disease mechanisms [5]. However, proteomics is not without its limitations, such as potential variability in protein detection and quantification, and sensitivity for low-abundance proteins, which need to be considered when designing proteomics experiments [5]. Several proteomic studies have been conducted, mostly in HD mouse models [6,7,8,9,10,11,12,13,14] or induced pluripotent stem cells (iPSCs) [15,16,17,18,19,20,21,22,23,24,25]. However, there are limited studies that comprehensively analyze the serum proteome of stage-specific HD patients [26,27,28,29,30,31]. 

Currently there are no studies investigating the proteomic profiles of Cypriot patients across the HD stages, a genetically unique population. The presence of founder effects may influence the expression and modulation of disease-related proteins, disease onset and progression and biomarker discovery with local and broader translational relevance, while enhancing our understanding of the molecular underpinnings of HD within this under-represented population. 

This study aims to explore the serum proteomic changes across asymptomatic, early symptomatic and symptomatic advanced HD using an untargeted LC-MS/MS-based approach, followed by bioinformatics-driven pathway analysis and biomarker analysis. Validation of key proteins using ELISA was also conducted. Our findings offer a new perspective on disease progression and identify promising candidates for early diagnosis and therapeutic intervention. 

## 2. Materials and Methods

**Figure 1:**
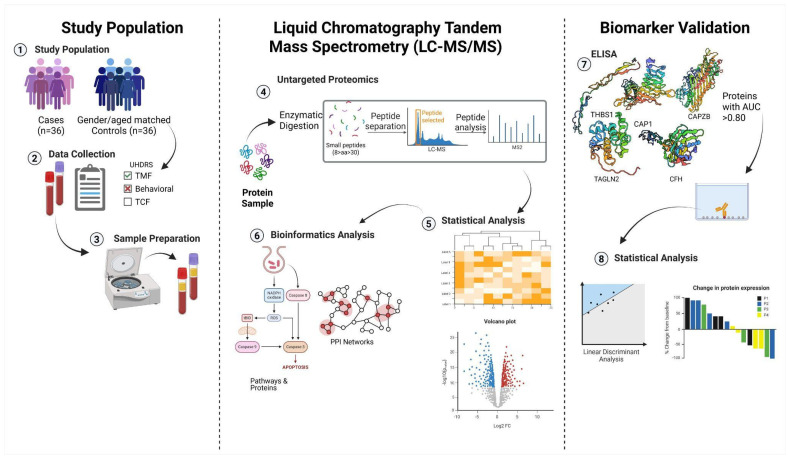
Schematic overview of proteomic and bioinformatics pipeline. Created in https://BioRender.com.

A schematic representation of the workflow methodology applied in this study is illustrated in Figure 1.

### 2.1. HD Patients and Controls 

This is a case–control study, consisting of (*n* = 36) individuals diagnosed with HD (asymptomatic (*n* = 18), early symptomatic (*n* = 8) and symptomatic advanced (*n* = 10)) and gender/age-matched controls (*n* = 36). Further detail regarding patient stratification can be seen in Section 2.3. Demographic and anthropometric data were collected and compared for cases vs. controls. 

In this study, all HD patients from The Cyprus Institute of Neurology and Genetics (CING), a referral center for HD in Cyprus, were invited to participate by their neurologist. The control group consists of HD family members without the pathological CAG trinucleotide expansion and carers of CING patients. Participant recruitment occurred between May 2021 and May 2022. Age of recruitment for HD individuals was between 18 and 75 years of age. Each HD individual was matched with a gender/age-matched control from HD families and caregivers (without the pathological CAG trinucleotide expansion). All subjects gave their informed consent for inclusion before participating in the study. The study was conducted in accordance with the Declaration of Helsinki and was reviewed and ethically approved by the Cyprus National Bioethics Committee with the ethical approval code EEBK/EΠ/2021/35. Written informed consent was obtained from all participants prior to study participation.

### 2.2. Data Collection 

Following consent, participants underwent an interview with a trained researcher, where a standard interviewer-administered questionnaire was given and anonymized, and the following data were obtained from HD patients and controls:

(i) Demographics: Sex, date of birth, birthplace and city of residence, birthplace of parents, family status (single, married, divorced or widowed) and occupation.
(ii) Medical and family history of HD: Presence and age of HD symptoms and family members who have HD.
(iii) Additional information: Height and weight prior to symptom onset in early symptomatic and symptomatic advanced stages.
(iv) Medical history, CAG repeats, treatments and other comorbidities obtained from the patient’s medical records or via self-reporting for controls.
(v) Lifestyle: Dietary intake, MD adherence, physical activity and smoking status.

### 2.3. Huntington’s Disease Assessment

The Unified Huntington’s Disease Rating Scale (UHDRS) [32] is frequently used to measure HD function and disease burden [32]; it is applied as both an endpoint for clinical research and as a screening tool for stratifying and selecting participants for inclusion in clinical trials and research studies [32]. A trained neurologist evaluated disease severity in HD patients using the UHDRS [32], to evaluate the four domains of motor, cognitive function, behavioral and functional capacity [32]. The motor domain focuses on the physical symptoms of HD, including oculomotor, dysarthria, involuntary movements (dystonia and chorea), bradykinesia, gait and balance [32]. The cognitive domain focuses on information processing (problem-solving abilities, memory and attention) [32]. The behavioral domain evaluates mood (depression, irritability, apathy), personality changes and behaviors (obsessions and compulsions) [32], while the functional capacity assessment evaluates how well an individual can perform daily activities (work, self-care, social interactions and finances) [32].

Patients were stratified based on their UHDRS scores. Asymptomatic patients had higher scores in TFC, demonstrating independence, while symptomatic and symptomatic advanced patients showed lower scores, indicating dependence and disability [32]. The total functional capacity (TFC) score indicates worsening in functional capacity [32]; it ranges from 13, indicating normal function and independence, to 0, total functional dependence [32]. Higher scores indicate overall better functioning in tasks related to occupation, domestic chores, finances, self-care and activities of daily living, while lower scores indicate increased impairment in the above-mentioned tasks [32]. 

### 2.4. Blood Collection and Serum Extraction

Blood samples were collected via the venipuncture method from the median cubital vein of each participant into two 9 mL EDTA tubes for plasma and 4 mL red-top silicon-coated tubes for serum. Plasma was obtained by centrifuging the EDTA sample at 3100 rpm for 5 min, while serum was extracted after allowing the blood to clot upright for 30–36 min, followed by centrifugation at 2000× *g* for 10 min at room temperature. Both plasma and serum aliquots were stored at −80 °C.

### 2.5. Sample Preparation

#### 2.5.1. High-Abundance Protein Depletion 

Serum samples were anonymized and shipped on dry ice to Creative Proteomics (New York, NY, USA), which has experience in offering high-throughput omics services. To improve detection sensitivity, high-abundance proteins (albumin, myoglobin, immunoglobulins (IgG), transferrin and haptoglobin) were depleted using equilibrated spin columns at room temperature. A 10 μL aliquot of serum was added to the resin slurry, mixed by inversion several times and incubated with gentle end-over-end rotation for 30 min at room temperature. Following centrifugation at 1000× *g* for 2 min, the resin was discarded. The resulting flow-through was filtered, and high-abundance proteins were effectively removed.

#### 2.5.2. Protein Digestion 

Protein digestion was performed on the filtered depleted samples. First, 5 μL of iced acetone was added, and then the samples were stored at −20 °C overnight and centrifuged at 12,000 rpm for 15 min; the resulting pellet was discarded. Proteins were redissolved in 50 mM ammonium bicarbonate and transferred to a Microcon device YM-10 (Millipore, Burlington, MA, USA). Buffer exchange was performed twice by centrifugation at 12,000× *g* at 4 °C for 10 min. Proteins were reduced with 10 nM DL-dithiothreitol (DTT) (Sigma, St. Louis, MO, USA) at 56 °C for 1h and alkylated with 20 mM Iodoacetamide (IAA) (Sigma, St. Louis, MO, USA) at room temperature in the dark for 1 h. Digestion was performed with trypsin (Promega, Madison, WI, USA) at a 1:50 enzyme-to-substrate ratio and incubated overnight at 37 °C. Extracted peptides were lyophilized to near dryness and resuspended in 20 μL of 0.1% formic acid (Sigma, St. Louis, MO, USA) prior to Nano LC-MS/MS analysis.

#### 2.5.3. Nano-Liquid Chromatography and Mass Spectrometry (LC-MS/MS) Analysis

Untargeted proteomics analysis was performed using the Ultimate 3000 nano-UHPLC system coupled with an Orbitrap Q Exactive HF mass spectrometer (Thermo Fisher Scientific, Waltham, MA, USA) equipped with an ESI nanospray ionization source. Peptide samples (1 μg) were loaded onto a PepMap C18 trap column (100 Å, 100 μm × 2 cm, 5 μm) and separated on an analytical column (PepMap C18, 100 Å, 75 μm × 50 cm, 2 μm). 

The mobile phase consisted of buffer A, comprising 0.1% Formic acid (Sigma, St. Louis, MO, USA) in water (Billerica, MA, USA), and buffer B, comprising 0.1% Formic acid (Sigma, St. Louis, MO, USA) in 80% Acetonitrile (Sigma, St. Louis, MO, USA). Chromatographic separation was achieved at a flow rate of 250 nL/min using a linear gradient over 95 min, specifically 2–8% buffer B (0–3 min), 8–20% buffer B (3–56 min), and 40–90% buffer B (91–95 min). 

Full MS scans were acquired in the *m*/*z* range of 300–1650 at a resolution of 60,000 (at 200 *m*/*z*); the automatic gain control (AGC) target for the full scan was set to 3 × 10^6^. MS/MS scans were acquired in the data-dependent acquisition (DDA) mode, using the top 20 most intense precursor ions, with the following parameters: (i) resolution: 60,000 at 200 *m*/*z*; (ii) AGC target: 3 × 10^6^; (iii) maximum injection time: 19 ms; (iv) normalized collision energy (NCE): 28%; (v) isolation window: 1.4 Th; (vi) charge state exclusion for unassigned, single charged, and charge >6 and (v) dynamic exclusion: 30 s. 

#### 2.5.4. Proteomic Data Analysis

Raw MS files (*n* = 72) were analyzed using MaxQuant (https://www.maxquant.org/) (version 1.6.2.6, accessed on: 19 December 2023) and searched against the *Homo Sapiens* protein database. The search parameters were as follows: (i) fixed modifications: carbamidomethylation and oxidation; (ii) variable modifications: oxidation of methionine; (iii) enzyme specificity: trypsin; (iv) a maximum of two missed cleavages allowed; (v) precursor ion mass tolerance: 10 ppm; and fragment ion mass tolerance: 0.5 Da. 

Differential protein expression analysis across HD stages vs. controls was performed using the unpaired *t*-test, and the false discovery rate (FDR) was applied for multiple testing. Total intensity normalization was applied; it assumes the total protein amount is similar across samples, and it scales the intensity values within each sample by a factor to equalize the total intensity across all samples.

Proteins with *p*-values < 0.05 and a fold change (FC) of >1.2 or FC < −1/1.2 were considered over- and under-expressed, respectively. Although the applied FC threshold is modest, it offers the advantages of (i) biological significance, as an FC > 1.2 represents a 20% change in protein abundance, which reflects a meaningful alteration in biological processes; (ii) statistical robustness, as a less stringent threshold allows identification of proteins exhibiting small but consistent and statistically significant differences, and the threshold applied assists in capturing a broader range of differentially expressed entities while maintaining a reasonable confidence level; and (iii) balanced sensitivity and specificity—an FC threshold of 1.2 is a practical compromise, enhancing the ability to detect biologically relevant changes while maintaining statistical confidence. 

#### 2.5.5. Quantification of CFH, CAPZB, CAP1, THBS1 and TAGLN2 in Serum via ELISA

ELISA quantification was performed by Creative Proteomics (New York, NY, USA), ELISAs were conducted to measure the levels of complement factor H (CFH), human (UniProt: P08603); capping actin protein of muscle Z-line subunit beta (CAPZB) (UniProt: P47756); cyclase associated actin cytoskeleton regulatory protein 1 (CAP1) (UniProt: Q01518); thrombospondin-1 (THBS1) (UniProt: P07996); and transgelin-2 (TAGLN2) (UniProt: P37802). All kits were in-house ELISAs with self-coated plates, and the antibodies used for coating are rabbit polyclonal antibodies. Serum samples from an independent HD cohort of asymptomatic (*n* = 7), symptomatic (*n* = 8), symptomatic advanced (*n* = 4) and (*n* = 19) healthy controls were measured and analyzed according to the manufacturer’s instructions. Samples were diluted with dilution factors of 1:5 prior to ELISA testing for CFH, CAPZB, CAP1, THBS1 and TAGLN2. Samples were assayed in triplicate and incubated at 37 °C, absorbance was measured at 450 nm. 

### 2.6. Statistical Analysis 

All statistical analyses were conducted using the STATA statistical software (versionSE16 StatCorp. 2007. College Station, TX, USA) and R studio (version 4.5.0, R Core Team, R Foundation for Statistical Computing, Vienna, Austria). Categorical variables (gender, BMI, clinical symptoms, inheritance pattern) were assessed using the chi-squared test, and continuous variables were assessed with the Wilcoxon signed-rank test (age) and *t*-test (age of onset) (AOO). Patients were stratified by disease stage for analysis of the UHDRS variables of total motor score (TMS), total behavioral score (TBS), TFC and independence scale (IS). In addition, Pearson’s correlation analysis was used to investigate correlations of TMS, TBS and TFC with IS for each HD stage. A *p* < 0.05 was statistically significant. 

A linear mixed-effects model (LMM) framework was used to assess protein expression differences between cases and gender/age-matched controls, as it accounts for repeated measures and nested data. Specifically, serum protein expression was measured across a panel of selected candidate biomarkers (CFH, CAPZB, CAP1, THBS1 and TAGLN2) in each participant. The LMMs were implemented using the *nlme* package for fitting and comparing linear and non-linear mixed-effects models and determining whether the within-group errors are correlated or have unequal variances [33]. The LMM was fitted with group, protein and interaction as fixed effects and subjects nested within replicates as a random intercept. LMMs consider unbalanced data and missing values and are an optimal choice for biomarker evaluation in complex, multi-factorial biological datasets [34,35]. In addition, the above-mentioned model accounts for both biological and technical sources of repeated measures. 

Model estimates were obtained using the *emmeans* package [36], which simplifies post hoc analysis, pairwise comparisons and estimation of marginal means from statistical models to visualize adjusted group-level differences across replicates. Pairwise comparisons between cases and controls were performed separately for each protein, and Benjamini–Hochberg false discovery rate correction was applied, aiding in avoiding Type I errors [37]. 

To quantify and determine the effect size of each protein for each HD stage vs. controls, Cohen’s *d* was applied, which measures effect size and quantifies the differences between cases and controls for each protein using the pooled standard error from EMMs [36]. Effect sizes were interpreted using standard thresholds of small (≤0.2), medium (≈0.5) and large (≥0.8). Data visualization was performed using *ggplot* [38], *ggpubr* [39] and tidyverse [40]. 

### 2.7. Bioinformatics Analysis Pipeline for Proteomic Data 

#### 2.7.1. Identification and Determination of the DEPs in Stage-Specific HD 

Post-processing of proteomic data was performed with MaxQuant (version 1.6.2.6, accessed on: 19 December 2023) to obtain a list of proteins for each HD stage. A total of *n* = 1638 proteins were recognized prior to differential expressed protein (DEP) identification. Statistical analysis was performed to obtain the FC and the *p*-value by applying the two-sample *t*-test for all the data. To identify the DEPs that were over- and under-expressed for each HD stage compared to controls, the FC cut-off was set with quantitative ratios of FC > 1.2, *p* < 0.05 (over-expressed) and FC < −1/1.2, *p* < 0.05 (under-expressed).

#### 2.7.2. Pathway and Gene Ontology Enrichment Analysis of Differentially Expressed Proteins in Stage-Specific HD 

The DEPs in each HD stage vs. controls were analyzed using Ingenuity Pathway Analysis (IPA) (Qiagen, Hilden, Germany) (www.qiagen.com, accessed on: 8 January 2024), a commercial bioinformatics software, and Metascape, an open-source tool (https://metascape.org, accessed on: 11 January 2024) [41]. IPA identified the canonical pathways and associated proteins across HD stages compared to controls; data input included the Uniport ID, FC and *p*-value for each HD stage vs. controls. Statistical significance for pathway enrichment was assessed using the right-tailed Fisher’s exact test, resulting in *p*-values for each pathway. Furthermore, IPA computes a z-score, reflecting the predicted activation state of each pathway based on the direction of expression changes in the dataset relative to known pathway behavior. A z-score near zero suggests no predicted activation or inhibition, while positive and negative values indicate pathway activation and inhibition, respectively. The z-score examines not only the presence of DEPs in a pathway but also the expected relationships and interactions among proteins. A heatmap was generated using the *pheatmap* package [42] to visualize pathway activity and expression profiles. 

To complement IPA analysis, gene ontology (GO) enrichment analysis was performed using Metascape, a web-based tool that integrates gene annotation and pathway enrichment data (https://metascape.org, accessed on: 11 January 2024) [41]. DEPs were analyzed for each HD stage compared to controls. Protein accession IDs were mapped to gene symbols with UniProt (https://www.uniprot.org, accessed on 11 January 2024) [43], and *Homo Sapiens* was selected as the reference organism. GO enrichment analysis was performed using biological process (BP), cellular component (CC) and molecular function (MF) libraries. Terms with a *p* < 0.05, a minimum gene count of 3, and an enrichment factor > 1.5 (the ratio of observed to expected counts) were retained. Enriched terms were clustered based on Kappa similarity scores, and hierarchical clustering was applied to group related terms with a similarity threshold of >0.3. The most statistically significant terms from each cluster were selected to represent the group. GO term clustering was generated using the *ClusterProfiler* [44] package. 

#### 2.7.3. Identification of Shared and Exclusive Proteins Across HD Stages

The Venn tool (https://bioinfogp.cnb.csic.es/tools/venny/index.html, accessed on: 15 January 2024) was used to identify the following overlapping and exclusive proteins within our study: (i) all proteins across HD stages vs. controls, (ii) DEPs identified across HD stages vs. controls and (iii) potential protein biomarkers across HD stages vs. controls. 

#### 2.7.4. Filtering and Evaluation of Biomarker Candidates for Huntington’s Disease Progression 

Potential HD stage-specific protein biomarkers were identified by biomarker filtering analysis using IPA, enabling the prioritization of candidate biomarkers from -omics datasets based on defined biological and clinical relevance. Parameters selected for analysis were (i) organisms: *Homo Sapiens*; (ii) biological fluid: blood, plasma and serum; and (iii) diseases: neurological diseases. Considering that all samples were derived from HD individuals at different disease stages, single-group biomarker comparison analysis was employed in our study. To evaluate the discriminative power of the identified biomarkers across disease stages, the area under the curve (AUC) was calculated using the auc() function. This provides a quantitative measure of each candidate’s performance in differentiating disease progression stages. 

#### 2.7.5. ELISA-Based Validation of Stage-Specific Biomarker Candidates in Huntington’s Disease 

Validation of stage-specific biomarker candidates (CFH, CAPZB, CAP1, THBS1 and TAGLN2), ELISAs were performed on an independent HD sample (*n* = 19) across all stages and (*n* = 19) gender/age-matched controls. Given the repeated measurements per subject and protein, LMMs were employed to model intra-individual variability [45]. LMMs are well-suited for hierarchical or nested data, enabling the inclusion of subject-specific random effects to control for intra-individual correlations and replicate variability [35] while improving statistical power and precision compared to traditional fixed-effects models [34,35]. 

#### 2.7.6. Analysis of Protein–Protein Interaction Networks Across Huntington’s Disease Stages 

To explore protein–protein interactions (PPIs) relevant to HD, we compiled a list of HD-associated proteins from two database sources, namely, DisGeNet v7.0 (https://disgenet.com/, accessed on: 9 May 2024) [46], from which 17 curated HD-associated genes (C0020179) were retrieved, and genome-wide association studies (GWAS) (accessed on: 9 May 2024) [47], from which 22 significant SNP-associated genes (*p* − 1 × 10^−6^) were obtained. The DEPs and the proteins measured and validated using ELISA were also integrated to construct PPI-HD networks using STRING v11.5 (https://string-db.org/, accessed on: 13 May 2024) [48]. The PPI-HD network was filtered based on a minimum combined confidence score of 0.4, a medium confidence threshold. The PPI-HD network constructed focused on two specific interaction types: (i) interactions between known HD-associated proteins and DEPs and identified protein biomarkers at each disease stage and (ii) interactions involving HTT proteins and their direct protein interactors.

## 3. Results

### 3.1. Demographics and Anthropometric Characteristics of Cohort 

This was a case–control study of *n* = 36 HD patients, specifically, patients in asymptomatic (*n* = 18), early symptomatic (*n* = 8) and symptomatic advanced stages (*n* = 10), along with *n* = 36 gender/age-matched controls. Demographic and anthropometric characteristics between cases and controls are shown in Supplementary Tables S1 and S2. 

Briefly, the mean age ± SD was 40.61 ± 10.51 years (*p* = 0.042) for asymptomatic patients, 51.71 ± 12.53 years (*p* = 0.014) for early symptomatic patients and 63.52 ± 9.48 years (*p* = 0.001) for symptomatic advanced patients. The mean AOO±SD was 46.14 ± 12.95 years and 55.71 ± 11.01 years for early symptomatic and advanced patients, respectively, with no statistical significance (*p* = 0.162).

**Figure 2:**
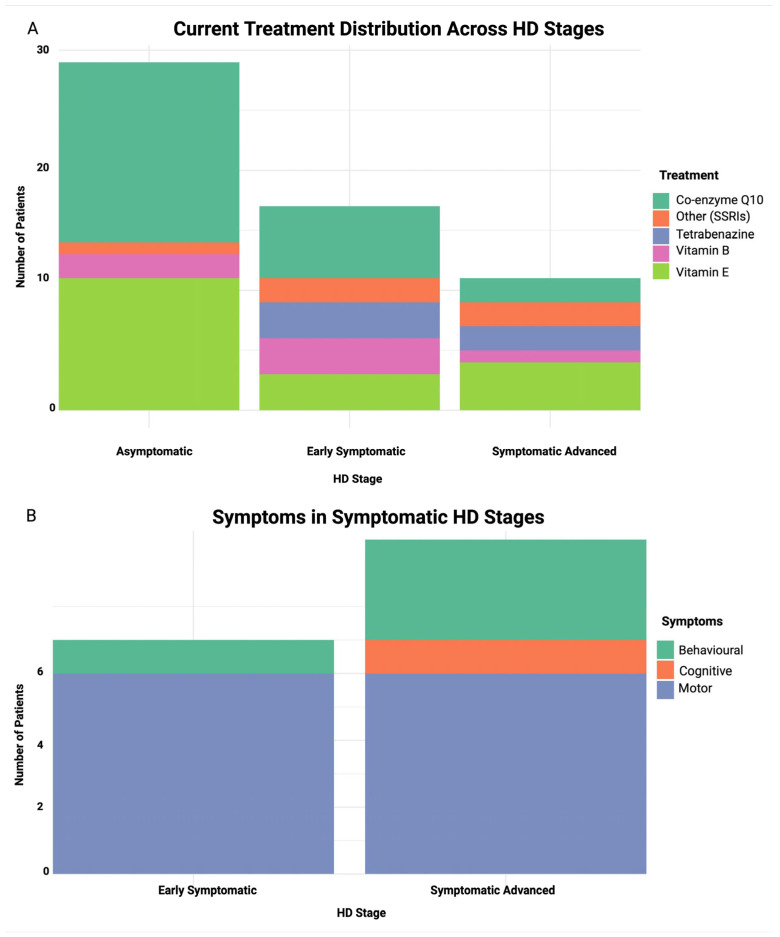
Stacked bar plot of treatment and symptom distribution across HD stages. (**A**) current treatment distribution across HD stages, (**B**) symptom distribution in early symptomatic and symptomatic advanced stages. X-axis represents HD stage and Y-axis represents number of patients in both plots.

Amongst early symptomatic and symptomatic advanced patients, motor impairment was frequent, at 86% and 60%, respectively, followed by behavioral impairment at 14% and 30%. Cognitive impairment was relatively uncommon and observed only in symptomatic advanced patients (10%) in our cohort (Figure 2). 

A slight predominance of maternal inheritance compared to paternal inheritance was observed across all groups: 53% vs. 47% in the asymptomatic group, 80% vs. 20% in the early symptomatic group and 67% vs. 33% in the symptomatic advanced group. Body mass index (BMI) was statistically significant (*p* = 0.007), declining in symptomatic stages, and weight and height were obtained prior to disease onset from either the patients’ next of kin or the patients’ medical records. 

We observed significant progression in motor, behavioral and functional impairment within our cohort (Table 1). The asymptomatic group demonstrated minimal motor signs, with a total motor score (TMS) of 5.4 ± 12.1, whereas symptomatic advanced patients exhibited severe motor impairment (47.2 ± 7.20; *p* < 0.0001). Regarding behavioral symptoms, the TBS increased from 8.0 ± 10.3 in asymptomatic individuals to 38.2 ± 21.9 in advanced HD, with a high prevalence of depression (in 64% of all patients) and dementia (in 88% of advanced cases). In terms of functional capacity, TFC declined sharply from 11.0 ± 4.1 in asymptomatic to 0.75 ± 0.7 in advanced individuals, paralleled by a decrease in estimated independence from 90 ± 5% to 15 ± 10% (*p* < 0.0001). Correlation analysis using Pearson’s correlation revealed that the TBS had a stronger association with loss of independence in early symptomatic patients compared to motor deficits (*p* = 0.0003), therefore suggesting that non-motor symptoms significantly contribute to functional decline, possibly during early disease stages. These findings highlight the critical interplay between behavioral impairments and loss of autonomy in HD progression, underscoring the need for comprehensive clinical assessment that extends beyond motor symptoms. 

Analysis of the current treatment regimen distribution across HD stages (Figure 2) revealed co-enzyme Q10 to be a common treatment in the asymptomatic stage (52%), and it decreases with disease progression from early symptomatic (35%) to symptomatic advanced stages (18%). Tetrabenazine usage was limited to symptomatic stages, aligning with its role in managing motor symptoms. Vitamin E, a well-studied antioxidant, was administered to patients across all stages, while SSRI use was mainly observed in the symptomatic stage groups. However, there was no statistically significant association between HD stage and treatment type (*p* = 0.131). 

### 3.2. Key Proteins and Identification of Differentially Expressed Proteins in Huntington’s Disease Pathogenesis

**Figure 3:**
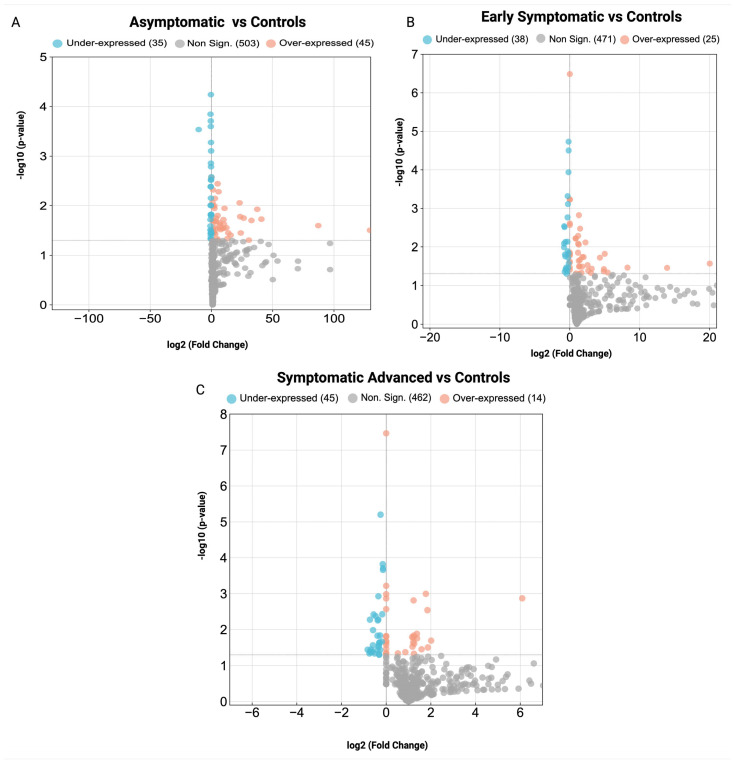
Volcano plots of differentially expressed proteins in stage-specific HD. (**A**) asymptomatic HD stage vs. controls, (**B**) early symptomatic HD stage vs. controls and (**C**) symptomatic advanced HD stage vs. controls. Non-significant proteins (gray), over-expressed proteins (orange) and under-expressed proteins (blue).

Untargeted proteomics analysis of serum samples identified *n* = 1638 proteins (Supplementary Table S3). DEPs were identified in the asymptomatic stage (80 DEPs), the early symptomatic stage (63 DEPs) and the symptomatic advanced HD stage (59 DEPs) compared to controls. The number of statistically significant over- and under-expressed DEPs for each group is shown in Table 2. To identify the DEPs for each HD stage compared to controls, the FC cut-off was set with quantitative ratios of FC > 1.2, *p* < 0.05 (over-expressed) and FC < −1/1.2, *p* < 0.05 (under-expressed). The volcano plots illustrating the DEPs for each HD stage are shown in Figure 3A–C, while the DEPS of each HD stage are in Supplementary Tables S4–S6, respectively. Pathway and GO enrichment analyses were also performed on DEPs with significant adj. *p*-values for each HD stage (Supplementary Tables S7 and S8). Furthermore, Venn diagrams were constructed to display all proteins identified across the HD stages (Supplementary Figure S1) and DEPs across all HD stages vs. controls (Supplementary Figure S2). 

### 3.3. Biological Pathways and GO Enrichment Terms in Stage-Specific Huntington’s Disease

**Figure 4:**
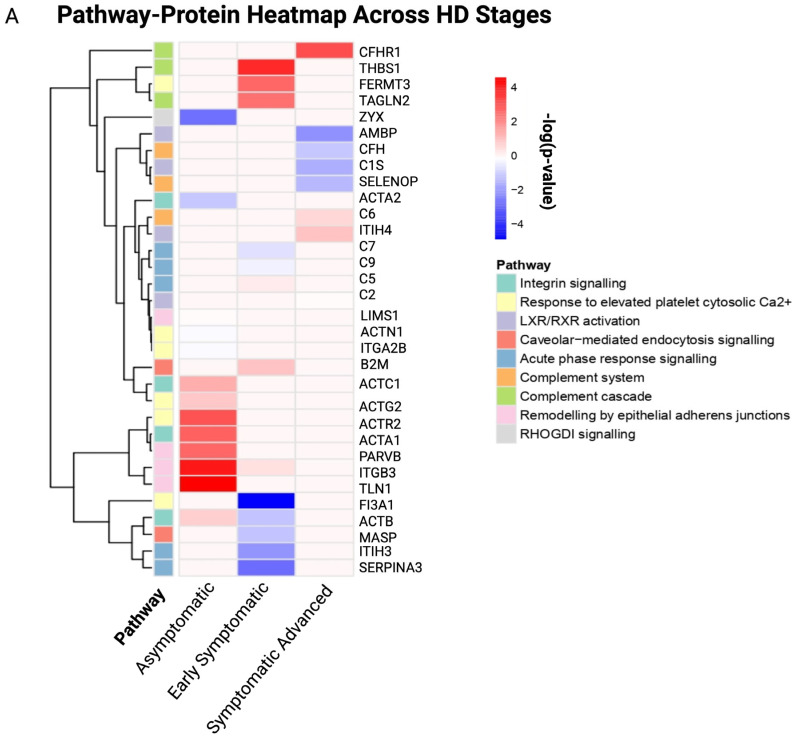

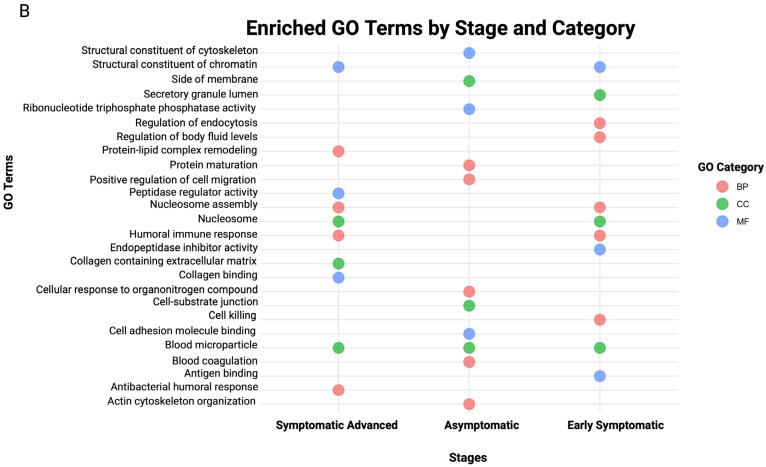
Pathway–protein heatmap and bubble plot of the top 10 GO terms for stage-specific HD. (**A**) Pathway–protein heatmap across HD stages, (**B**) bubble plot of the top 10 GO terms across HD stages. The x-axis represents the HD disease stages, and the y-axis represents identified proteins for each pathway, as indicated by the colored cells and figure legend. The Y-axis represents the proteins identified for each pathway, and the X-axis represents the canonical pathways present in each HD stage. A positive correlation is indicated by a dark red color, whereas no correlation is indicated by light pink.

It is possible to obtain a deeper understanding of the biological roles of the DEPs via enrichment analysis. Therefore, in the existing study, pathway enrichment analysis and GO were performed to reveal the biological pathways and processes, molecular functions and cellular components affected across HD stages compared to controls. Pathway–protein heatmaps across HD stages are illustrated in Figure 4A. 

Asymptomatic DEPs were predominantly associated with integrin signaling (*p* = 1.32 × 10^−15^), response to elevated platelet cytosolic Ca^2+^ (*p* = 2.51 × 10^−18^), remodeling by epithelial aderens junctions (*p* = 3.52 × 10^−20^) and RHOGDI signaling (*p* = 2.78 × 10^−20^), highlighting cytoskeleton instability as an early hallmark. In contrast, early symptomatic DEPs were associated with acute phase response signaling (*p* = 3.71 × 10^−5^), response to elevated platelet cytosolic Ca^2+^ (*p* = 5.47 × 10^−11^), complement cascade (*p* = 1.08 × 10^−5^), caveolar-mediated endocytosis signaling (*p* = 6.29 × 10^−5^) and the complement system (*p* = 5.75 × 10^−8^). The symptomatic advanced stages were associated with LXR/RXR activation (*p* = 1.93 × 10^−3^), the complement system (*p* = 3.29 × 10^−9^), response to elevated platelet cytosolic Ca^2+^ (*p* = 2.22 × 10^−3^), acute phase response signaling (*p* = 2.25 × 10^−6^) and the complement cascade (*p* = 6.27 × 10^−9^). 

The complementation of GO annotation with pathway analysis provides a holistic outcome of altered biological processes, molecular functions and cellular components related to the statistically significant DEPs for each HD disease stage. GO terms emphasized dysregulation of immune response, vesicular trafficking and lipid metabolism. The top 10 GO terms of each GO library were used to generate a bubble plot (Figure 4B); furthermore, the full list of GO terms in each HD stage is provided in Supplementary Tables S9–S11.

### 3.4. Potential Protein Biomarker Candidates for Stage-Specific Huntington’s Disease

**Figure 5:**
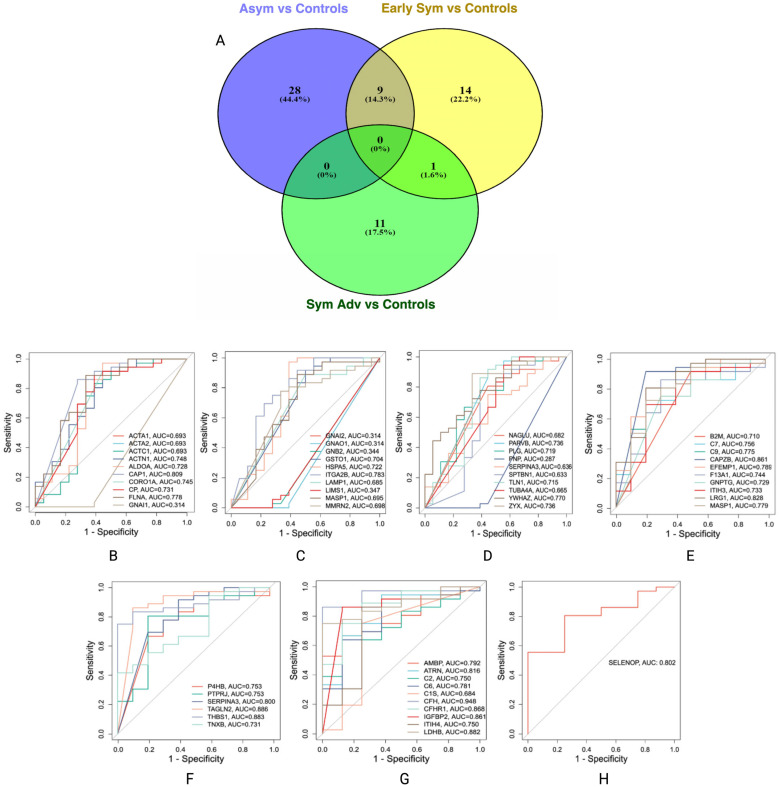
Venn diagram of exclusive and shared protein biomarkers and AUCs of protein biomarkers for stage-specific HD. (**A**) venn diagram of the exclusive and shared protein biomarkers identified with biomarker comparison analysis for stage-specific HD. (**B**–**D**) AUCs for asymptomatic HD stage vs. control. (**E**,**F**) AUCs for early symptomatic HD stage vs. control and (**G**,**H**) AUCs for symptomatic advanced HD stage vs. control. A maximum of ten biomarkers were included in each AUC; therefore more than one plot is presented for each HD stage comparison.

Biomarker filter analysis revealed several potential protein biomarkers across HD stages: asymptomatic (*n* = 37), early symptomatic (*n* = 24) and symptomatic advanced (*n* = 12); a comprehensive list of the candidate biomarkers is provided in Supplementary Table S12. Single-group biomarker comparison analysis revealed overlapping and exclusive protein biomarkers across HD stages. Exclusive protein biomarkers include *n* = 28 for asymptomatic, *n* = 14 for early symptomatic and *n* = 11 for symptomatic advanced HD stages (Supplementary Table S13). Nine shared biomarkers, namely, ACTB, C5, FERMT3, GP1BA, HSP90B1, ITGB3, MASP1, PDIA3 and SERPINA3, were common between the asymptomatic and symptomatic advanced HD stages, and the PLXDC2 protein was shared between the early and symptomatic advanced HD stages (Figure 5A).

To investigate and evaluate the diagnostic potential of these biomarkers, the area under the curve (AUC) was calculated for each protein relative to controls; only the exclusive biomarkers of each HD stage were selected for AUC analysis, as these represent the most promising candidates for stage-specific biomarkers. Proteins with an AUC ≥ 0.8 (Figure 5B–H) were considered biomarkers of interest warranting further investigation: adenylyl cyclase associated proteins 1 (CAP1) (*AUC* = 0.809), F-actin capping protein subunit beta (CAPZB) (*AUC* = 0.861), transgelin 2 (TAGLN2) (*AUC* = 0.886), thrombospondin 1 (THBS1) (*AUC* = 0.883) and complement factor H (CFH) (*AUC* = 0.948). These findings suggest a panel of promising stage-specific biomarkers for HD progression that may have diagnostic and therapeutic implications; therefore, an independent case–control cohort was used to validate the identified potential biomarkers. 

### 3.5. Independent Validation of Proteomic Candidates in Huntington’s Disease

**Figure 6:**
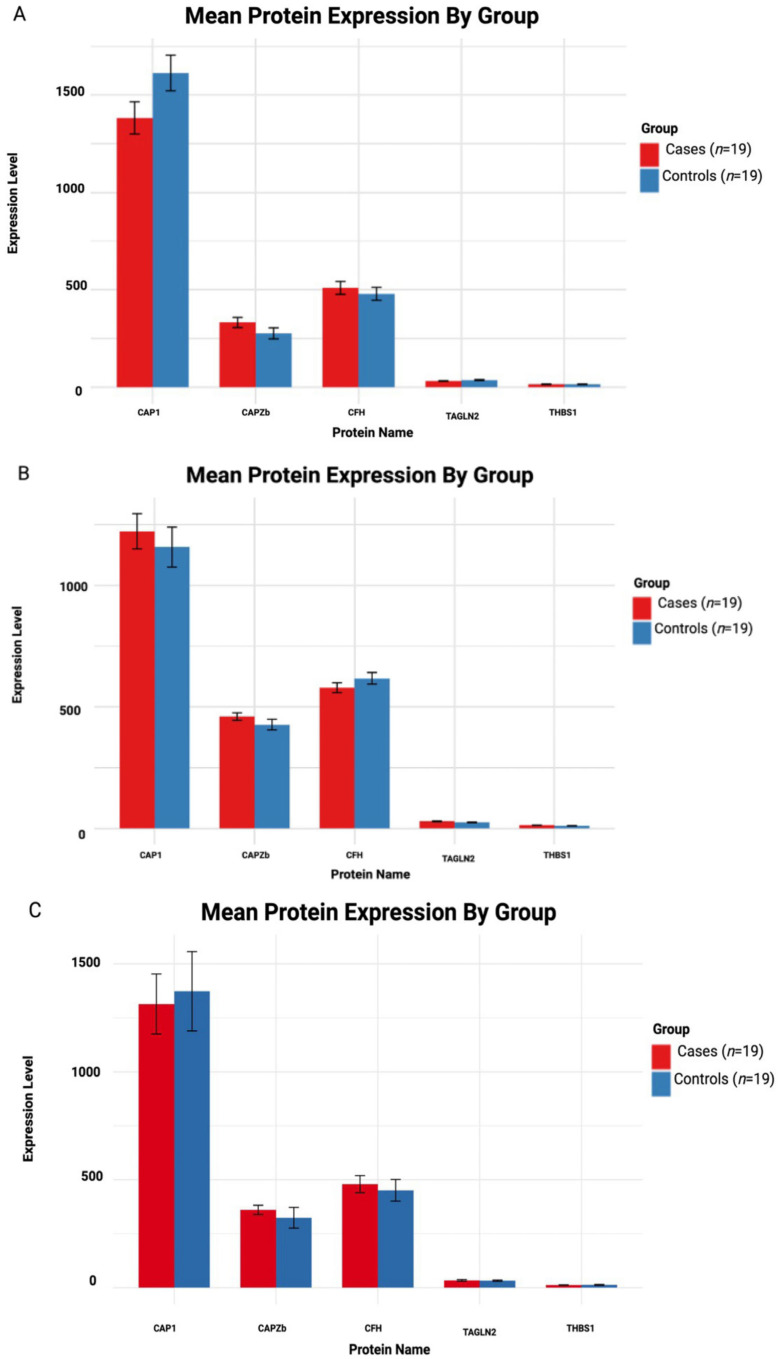
Mean protein expression in HD cases and controls. (**A**–**C**) Asymptomatic, early symptomatic and symptomatic advanced vs. controls, respectively. The case group (red) and control group (blue). There was a total of *n* = 19 cases and *n* = 19 controls.

To explore the identified potential protein biomarkers, we generated an LMM based on quantified protein expression in serum samples from individuals across the three HD disease stages vs. gender/age-matched controls. For each comparison, estimated cases vs. control group differences, standard errors, adjusted *p*-values and Cohen’s *d* effect sizes were calculated (Table 3). The mean protein expression for each HD stage vs. controls is illustrated in Figure 6A–C.

#### 3.5.1. Asymptomatic HD vs. Controls

CAP1 emerged as the most statistically significant altered protein in the asymptomatic stage; it demonstrated a decrease in HD cases compared to controls (Figure 6A) (estimate = −230.76, *SE* = 61.45, *p* = 0.0027), with a large effect size (*d* = −5.31). Therefore, decreased CAP1 may precede clinical onset and lead to a potential early disease biomarker. CAPZB and CFH proteins were modestly elevated in cases compared to controls (*d* = 1.28 and *d* = 0.69, respectively), while differences did not reach statistical significance (*p* > 0.05); these proteins could be worth investigating and may be biologically relevant in large cohorts. However, TAGLN2 (*d* = −0.111) and THBS1 (*d* = −0.001) exhibited little to no group differences, indicating negligible changes at the asymptomatic disease stage. 

#### 3.5.2. Early Symptomatic HD vs. Controls

The CAP1 protein displayed a non-significant increase in cases (Figure 6B), although a large effect size (*d* = 1.58) was obtained, suggesting sample size limitations may have masked statistical significance. A moderate to large effect (*d* = 0.80) was observed for the CAPZB protein, where a non-significant increase in cases (*p* = 0.576) was observed. Symptomatic cases displayed a decrease in CHF compared to controls (estimate = −38.53, *d* = –0.93), with no statistical significance (*p* = 0.518). In addition, TAGLN2 (*d* = 0.101) and THBS1 (*d* = 0.0668) remained largely unchanged, with no observed differences at the early symptomatic disease stage. 

#### 3.5.3. Symptomatic Advanced HD vs. Controls

CAP1 displayed a medium effect (*d* = −0.743) with a non-significant (*p* = 0.69) decrease compared to controls (Figure 6C). Medium to small effect sizes were observed for CAPZB and CFH proteins (*d* = 0.454 and *d* = 0.362, respectively), while TAGLN2 (*d* = 0.454) and THBS1 (*d* = −0.008) continued to display no minimal differences between cases and controls.

**Table 3:** Pairwise comparison and effect sizes of potential protein biomarkers in stage-specific HD vs. controls.

Group Name	Protein	Estimate	SE	adj. *p*-Value	Mean Cases	Mean Control	Cohen’s *d*
Asymptomatic HD vs. Controls	CAP1	−230.76	61.45	0.0027 *	1382.10	1612.86	−5.31
	CAPZB	55.81	61.45	0.381	331.94	276.12	1.280
	CFH	30.27	61.45	0.631	509.47	479.19	0.699
	TAGLN2	−4.81	61.45	0.938	31.43	36.24	−0.111
	THBS1	−0.056	61.45	0.992	14.56	14.61	−0.001
Symptomatic HD vs. Controls	CAP1	65.08	58.11	0.281	1222.53	1157.44	1.580
	CAPZB	33.20	58.11	0.576	460.65	427.45	0.808
	CFH	−38.53	58.11	0.518	579.30	617.84	−0.938
	TAGLN2	4.15	58.11	0.944	29.15	25.00	0.101
	THBS1	2.74	58.11	0.963	13.72	10.98	0.066
Symptomatic Advanced HD vs. Controls	CAP1	−59.04	112.30	0.617	1314.06	1373.11	−0.743
	CAPZB	36.05	112.30	0.759	360.14	324.09	0.454
	CFH	28.79	112.30	0.800	479.70	450.90	0.362
	TAGLN2	1.89	112.30	0.987	33.71	31.81	0.024
	THBS1	−0.70	112.30	0.995	12.44	13.57	−0.008

SE: Standard Error. Cohen’s *d* effect size: small (*d* = 0.2), medium (*d* = 0.5) and large effect sizes (*d* ≥ 0.8); *p*-values > 0.05 were statistically significant, as indicated by * and bold.

### 3.6. Protein–Protein Interaction Networks and Functional Context for DEPs of Stage-Specific HD

**Figure 7:**
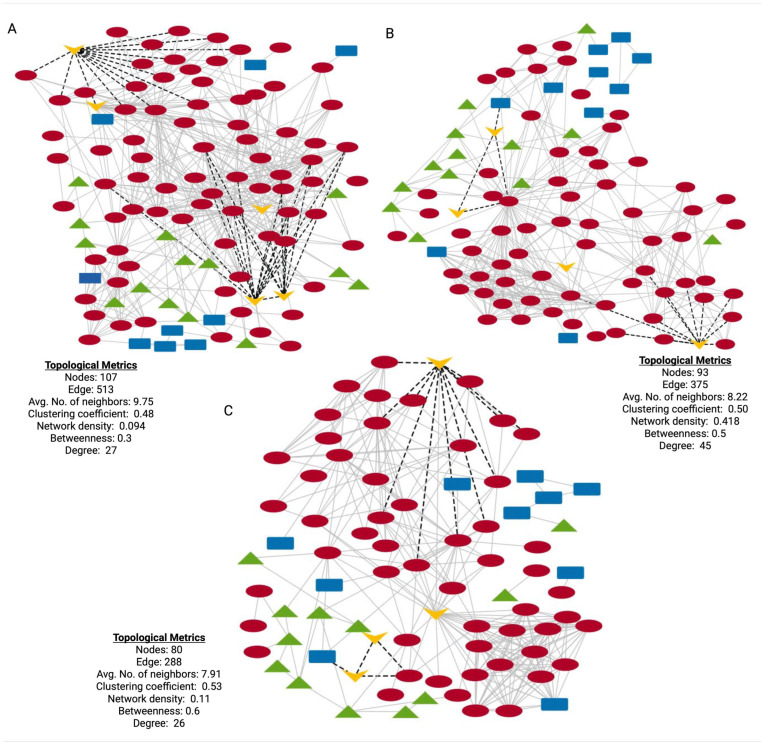
PPI networks of HD-associated genes, DEPs and candidate biomarkers across HD stages. (**A**–**C**) HD-PPI networks for asymptomatic, early symptomatic and symptomatic advanced vs. controls, respectively. Blue rectangular nodes: GWAS-HD-associated genes; green triangular nodes: DisGeNet-HD-associated genes; red circular nodes: DEPs for each specific HD stage; yellow V-shaped nodes: validated biomarkers. Nodes represent proteins and edges represent protein–protein interactions.

To investigate the functional relationships among DEPs in HD, stage-specific protein–protein interaction (PPI) networks were constructed using STRING (https://string-db.org/, accessed on: 13 May 2024) [48]; these networks provide insight into the molecular interactions and biological processes potentially dysregulated during disease progression. In the HD-PPI networks (Figure 7A–C), HTT was the central connecting node, interacting with multiple proteins involved in key cellular and biological functions, including mitochondrial function (AIFM1, MAOB), dopamine synthesis and neuroprotection (GDNF), cytoskeleton structure and intercellular signaling (ACTN1, ACTB, CAP1 and CAPZB) and complement activation (CFH, MASP1 and COLEC10). Further research is required to explore these proteins and pathways as potential therapeutic targets in delaying disease onset and progression.

## 4. Discussion

The widespread use of -omics-based approaches has significantly enhanced our understanding of rare diseases. Genomic studies [49,50,51,52] identified several genetic modifiers (FAN1, MSH3, PMS1 and PMS2) influencing disease onset and progression, providing critical insights into disease heterogeneity. Transcriptomic studies [19,53,54,55,56,57,58,59,60] have assisted in elucidating gene expression changes underlying cellular dysfunction in HD, while proteomic and metabolomic studies [57,61,62,63,64,65,66] are comparatively less common, but are emerging as valuable methods for identifying disease biomarkers and therapeutic targets. 

Proteomics is a promising approach due to its ability to capture dynamic protein-level changes driven by multiple biological factors; the proteome composition is influenced by multiple factors (genetic, environmental, developmental and physiological factors) [4]. In the present study, we conducted untargeted proteomic analysis across asymptomatic, early symptomatic and symptomatic advanced HD stages compared to gender/age-matched controls. Our aim was to identify DEPs, map associated biological pathways and highlight candidate biomarkers for disease detection and progression monitoring. 

The complement system is part of the innate immune system and viewed as the connection between innate and adaptive immune responses [67], consisting of >20 distinct proteins circulating in the blood and tissue, with roles in opsonization for enhancing phagocytosis of antigens, chemotaxis for attracting neutrophils and macrophages, and cell lysis for rupturing membranes of foreign cells [67]. Most complement proteins and receptors are expressed by astrocytes, microglia and neurons [67]. There is significant emerging evidence that the complement system has a role in ND development and progression. 

In HD, the complement system is activated by peptides such as mHTT; several complement factors (C1q, C4 and C3) are expressed in the striatum [67]. Studies have shown C1q, C3 and C4 to be over-expressed in post-mortem human and mouse brains, with C1q and C3 also being involved in excessive synaptic loss [67]. Early and advanced HD stages demonstrated an increased involvement of immune-related and metabolism-related pathways shown to have roles in NDs. However, it currently remains unclear which factors initiate the detrimental activation of the complement system [67]. While it normally serves to protect the brain from infection and assist in debris clearance, chronic activation or dysregulation in the central nervous system (CNS) leads to neuroinflammation, synaptic loss and neuronal cell death [67,68]. 

The complement system and cascade, along with C5, C7, C9 and MASP1 proteins, were identified in the early symptomatic and symptomatic advanced groups. Complement component (C5) and MASP1 are part of the lectin pathway: C5 plays a role in membrane attack and pro-inflammatory inflammation [69,70], and mannan-binding lectin serine protease 1 (MASP-1) involves MASP2 and MASP3 activation and cleavage of C4 and C2 into fragments to form a C3-convertase [71]. C7 is part of the classical pathway, with a role in cell lysis and death [72] and C9 is part of the alternative pathway, with a role in membrane attack [73]. The identification of this pathway in HD is consistent with previous studies [68,74,75]. 

A study by Singhrao et al. [68] investigated complement activation and biosynthesis in post-mortem HD brains (*n* = 9) and healthy controls (*n* = 3); the striatum, neurons, myeline and astrocytes were stained with antibodies to C1q, C4, C3, iC3b-neoepitope and C9-neoepitope, and a normal striatum presented with no complement staining [68]. Moreover, activators of the classical pathway, C1q C chain, C1r, C4, C3, complement regulators, C1 inhibitor, clusterin, MCP, DAF and CD59 were highly expressed in HD brains compared to controls. Increased expression of C5a and C3a receptors was observed in the HD caudate, resulting in overall neuronal and synaptic loss [68]. In addition, complement mRNA in normal brain tissue was 2–5-fold lower compared to that in the HD striatum, while C3 and C9 mRNAs were expressed by reactive microglia in the HD internal capsule. Moreover, the study observed that complement proteins produced by reactive microglia were activated on neuronal membranes, contributing to neuronal necrosis and a pro-inflammatory response [68].

In Alzheimer’s disease (AD), complement activation and inflammation occur and provide justification for investigating the effects of anti-inflammatory treatments in AD; for the same reasoning, therapy with anti-inflammatory agents or anti-complement agents may be of possible benefit in HD, whereas it is quite likely that treatment would have to be initiated at early disease onset, prior to neuronal loss. Although this does not address the underlying cause of HD, effective interventions may slow neuronal loss in an otherwise fatal disease. 

The liver X receptors (LXRs) are nuclear receptors with roles in lipid metabolism and transport and the inflammatory response within the CNS [76]. There are two isoforms: LXR_α_ is predominately expressed in the liver, kidney, small intestines and adipose tissue for peripheral lipid metabolism, and LXR_β_ is predominately expressed in the brain [77]. In NDs there is an unfavorable response due to limited neuronal regeneration in the CNS; therefore, due to the LXRs’ involvement in lipid metabolism and the inflammatory response, there is growing interest in investigating the role of LXR and LXR-related therapies in NDs. Previous studies of Parkinson’s Disease (PD) [78,79,80], AD [80,81,82] and HD [77,80,83,84] investigated the role of LXR/RXR in these NDs. 

A study by Leon et al. [83] observed dysregulation of cholesterol homeostasis in HD mouse models and patients and decreased CYP46A1 expression, a brain-specific enzyme essential for cholesterol turnover in the CNS [77,83]. CYP46A1 knockdown in the mouse striatum resulted in HD-like striatal neuronal degeneration, whereas CYP46A1 over-expression in the striatum of mice decreases the severity of neuronal cell loss and HTT aggregates, resulting in improved motor function [77,83]. However, it remains unclear whether the mechanism involves CYP46A1-dependent production of 24S-hydroxycholesterol (24-OHC) and activation of LXRs, although 24-OHC overproduction in CYP46A1-expressing mice was unable to affect LXR activation in the brain and liver. Nevertheless, disruption of cholesterol homeostasis in the brain may be a component in disease progression, as cholesterol has a role in synaptogenesis, and neurite outgrowth and neurotransmitter release are impaired in HD. 

Integration of proteomic and lipidomic networks offers a promising approach for detecting and monitoring HD, as the protein and lipid profiles are altered across HD stages. Various protein biomarkers have exhibited promise in HD, including mHTT, directly reflecting the underlying mutation and neurofilament light chain (NfL), a marker of axonal damage that is associated with disease progression [85,86,87]. On the other hand, increasing evidence points to dysregulation of cholesterol metabolism in HD, increasing interest in 24-OHC [85,88]. Furthermore, dysregulation of 27-hydroxycholesterol, oxysterol ratios and other lipids has also been reported in HD patients and animal models [85,87]. Therefore, multi-omics integration of protein and lipid biomarkers may provide a more holistic and dynamic insight into HD pathology, resulting in improved diagnostic sensitivity and monitoring of treatment response. This multi-omics approach has been implemented in AD [89], with phospholipids, triglycerides, sphingolipids and cholesterol esters correlated with AD risk and with pathway analysis indicating involvement of the immune response and lipid metabolism [89]. In contrast, pathway analysis of proteins demonstrated their involvement in positive regulation of cytokine production, neutrophil-mediated immunity and the humoral immune response. The following illustrates that tightly regulated lipids and proteins are drivers in lipid homeostasis and innate immunity, strongly associated with AD pathology [89].

In our study LXR/RXR activation and the AMBP and ITIH4 proteins were identified in the symptomatic advanced group. AMPB plays a role in brain physiology, and under pathological conditions its expression becomes altered [90]. AMBP is a glycoprotein, with roles as an antioxidant and a tissue repair protein, a regulator of inflammatory processes and a protease inhibitor. Studies have observed that AMBP-1 down-regulation is involved in AD [90]; this leads to an overactivation of the alternative complement pathway and increased inflammation [90]. However, AMBP does not seem to have a direct role in HD. Further research is required to understand the role of AMBP in NDs and potential therapeutic targets. ITIH4 is considered to have anti-inflammatory and protease-inhibitory properties [91]. Previous studies of amyotrophic lateral sclerosis (ALS) [92] and AD [93] have demonstrated increased ITIH4 expression during early disease stages in ALS patients compared to controls, while in AD, it was observed to be increased in the hippocampus, thalamus and cerebral cortex [92,93]. However, the exact mechanism of action of ITIH4 in HD remains unclear, although based on the above studies, it is likely involved in modulating inflammatory responses. However, further studies are required to investigate whether ITIH4 expression is increased or decreased in HD. 

Table 4 summarizes the discussed DEPs and their biological functions and findings in previous studies. 

Our study identified possible biomarkers, namely, CAP1 and CAPZB, which have been shown to be potential early detection and general disease biomarkers. However, further validation in larger, independent HD cohorts and comparison with other NDs are necessary to assess the specificity and sensitivity of CAP1 and CAPZB as HD biomarkers. Longitudinal studies and functional assays could help clarify whether CAP1 and CAPZB play a causal role in HD pathophysiology or represent a downstream consequence of broader cellular dysfunction, which may be translated to the clinic for diagnostic and prognostic purposes.

CAP1, a multidomain actin-binding protein, regulates cytoskeletal dynamics and participates in several key cellular processes, including vesicle trafficking and metabolism signal transduction pathways. CAP1 is expressed in the nervous system (NS), where it contributes to neuronal development and synaptic function via the modulation of actin filament organization [96]. CAP1 expression is involved in embryonic neuronal differentiation, while CAP2 is predominantly expressed in the cortex and hippocampus [96]. 

A previous study by Chang et al., 2012 [97], reported decreased CAP1 expression in peripheral leukocytes from HD carriers and those with HD, as well as in transgenic HD mouse models, compared to their respective controls. The study implicates CAP1 as a potential candidate peripheral biomarker for early disease detection. In the study, AHCY1, ACO2, OXCT1 and CAP1 were significantly under-expressed; these proteins were associated with oxidative stress response and metabolic pathways [97]. Although the role of CAP1 in HD has not been thoroughly studied in comparison to other more established contributors to HD pathogenesis, there may be a few potential connections between CAP1 and HD, as it is well established that mHTT disrupts actin cytoskeleton dynamics [96], resulting in impaired axonal transport and synaptic dysfunction. This suggests a possible mechanistic association between CAP1 expression and HD pathogenesis. 

Our findings partially align with these observations, as CAP1 expression was decreased in the asymptomatic group, supporting its potential as an early-stage biomarker. However, its expression increased in the early symptomatic group and declined in the advanced stage, suggesting variability in its usefulness as a progression marker. This fluctuation highlights the need for further investigation of CAP1 in HD, raising the possibility that CAP1 may be a more reliable marker for early detection than for tracking disease progression. 

CAPZB, an actin cytoskeleton regulator, directly binds to the barbed end of F-actin β-tubulin, thereby regulating actin polymerization and filament length [91], which are essential for numerous cellular processes, such as cell morphology and motility. In neuronal cells, actin dynamics are vital for neurite extension, synaptic function, and neuronal structure and function [98]. CAPZB dysfunction may lead to impaired actin filament growth, potentially contributing to the neurodegenerative processes seen in HD [98].

A study by Vanderburg et al. [98] investigated CAPZB2 expression, a variant of CAPZB, in post-mortem HD brains. The study found that silencing CAPZB2 in hippocampal neurons resulted in shortened neurites with abnormal growth cones, mirroring cytoskeletal abnormalities observed in HD. Furthermore, decreased neurite length was observed in both AD and HD, suggesting a shared neurodegenerative pathway involving cytoskeleton disruption. Additionally, Western blot analysis revealed disease and region-specific alterations in CAPZB2 protein expression, emphasizing its potential involvement in HD pathology [98].

In our study, CAPZB expression illustrated a large effect size and was consistently elevated across all HD stages. This robust and stable expression pattern highlights CAPZB as a promising candidate biomarker for HD and warrants further investigation into its role as a potential general disease marker.

Among our most notable findings is the early under-expression of CAP1 in asymptomatic HD, correlating with the existing literature on its role in neuronal development and its decreased expression in leukocytes obtained from patients [97]. In contrast, CAPZB was observed to be consistently elevated across HD stages, reinforcing the notion that actin cytoskeleton remodeling occurs early in HD pathogenesis. This outcome strengthens the evidence that neuroinflammation is a secondary driver of neurodegeneration.

Strengths and Limitations

The present study has several strengths and limitations. With regard to the strengths, (i) to the best of our knowledge, this is the first study investigating the proteomic profiles of Cypriot HD patients across HD stages; (ii) DEPs and biological pathways were identified for each HD stage, providing further insight into the proteins and pathways dysregulated in HD; (iii) serum was used as our biological fluid, as there is a growing interest in using plasma or serum to identify potential disease-specific biomarkers, as it is less invasive than CSF; (iv) an untargeted proteomic approach was used for simultaneous measurement and comparison of thousands of proteins within biological samples without prior knowledge, allowing for comprehensive analysis of protein changes across disease stages [99]; (v) proteomics facilitates drug development and biomarker discovery by providing a comprehensive map of protein interactions associated with disease pathways [4]; and (vi) potential biomarkers were validated with ELISA in an independent case–control cohort. Our study limitations include the following: (i) A relatively small sample size was used: as HD is a monogenic disease and Cyprus is a small island, this ultimately affects the study sample size. Additional factors include patients and carers missing appointments, not wanting to be reminded of their disease progression or having no hope for a drug treatment during their lifetime [4], which prevents patients from visiting neurologists or taking part in research. Although we have a small sample size, we managed to obtain significant proteins, pathways and biomarkers. (ii) Although ELISA validation analysis was performed, further in-depth validation is required in larger and more diverse HD cohorts and in other NDs to determine the specificity and sensitivity of CAP1 and CAPZB as potential HD biomarkers. (iii) Technical limitations are related to protein extraction, separation, identification and quantification, as each step may introduce bias and variability, potentially resulting in data reliability issues, sensitivity for low-abundance proteins [100] and difficulty in result verification, as the complexity of proteomic data can make it challenging to validate results [99].

Future Directions

Future work should include in vitro assays of neuronal cell models with either siRNA knockdown or CAP1/CAPZB over-expression to investigate their impact on the actin cytoskeleton, synaptic structure and function and oxidative stress. External validation of DEPs and biomarker panels should be performed on HD patients using public HD datasets, and biomarker analysis should be performed on other NDs (AD, PD and ALS) to assess the specificity and sensitivity of CAP1 and CAPZB. Longitudinal studies tracking biomarker and DEP changes over time as patients progress from one disease stage to the next should be conducted. Lastly, sample size and diversity should be increased through a multicenter or international collaboration with CHDI and ENROLL-HD.

## 5. Conclusions

Ultimately, this study enhances our understanding of the molecular alterations in HD, paving the way for future research that may lead to improved strategies for managing and treating the disease. This study elucidates the serum proteomic alternations across the HD stages, offering a high-resolution interpretation of disease-associated changes from the asymptomatic stage through to the symptomatic advanced stage. Key molecular disruptions were observed in cytoskeletal remodeling and regulation, with CAP1 and CAPZB emerging as candidate stage-specific biomarkers. In addition, lipid dysfunction and the immune system may play a role in HD disease progression, as these biological pathways have been implicated in several neurodegenerative diseases. These findings highlight the potential for serum-based proteomic biomarkers to facilitate earlier diagnosis, track disease progression and inform targeted interventions. Future longitudinal and mechanistic studies are required to validate these markers and translate them into the clinical setting, paving the way for precision diagnostic tools and targeted interventions, potentially transforming the clinical management of this devastating disease.

## Figures and Tables

**Table 1:** Statistical analysis of UHDRS characteristics of Cypriot HD patients.

UHDRS Characteristics of Cypriot HD Patients					
Variable		HD (*n* = 36)	Asymptomatic (*n* = 18)	Early Symptomatic (*n* = 10)	Symptomatic Advanced (*n* = 8)
**TMS (0–124)**	Mean (SD)	21.38 (20.19)	5.4 (12.1)	29.4 (6.94)	47.2 (7.20)
**TBS (0–224)**	Mean (SD)	18.66 (18.06)	8.0 (10.3)	22.2 (10.60)	38.2 (21.9)
**IS (0–100%)**	Mean (SD)	65 (5.27)	90 (5)	58 (8)	15 (10)
**TFC (0–13)**	Mean (SD)	7.08 (5.27)	11 (4.14)	5.1 (2.13)	0.75 (0.70)
**Behavioral Milestones**					
***Confused***	N (%)				
***No/Yes***		29 (64)/7 (19)	16 (89)/2 (11)	9 (90)/1 (10)	4 (50)/4 (50)
***Demented***	N (%)				
**No/Yes**		23 (64)/13 (36)	10 (56)/8 (44)	6 (60)/4 (40)	1 (13)/7 (88)
***Depressed***	N (%)				
**No/Yes**		13 (36)/23 (64)	10 (56)/8 (44)	1 (10)/9 (90)	2 (25)/6 (75)
***Requiring SSRI’s***	N (%)				
**No/Yes**		13 (36)/23 (64)	10 (56)/8 (44)	0/10 (100)	3 (38)/5 (63)

TMS: Total Motor Score; TBS: Total Behavioral Score; IS: Independence Scale; TFC: Total Functional Capacity; SD: Standard Deviation; SSRIs: Selective Serotonin Reuptake Inhibitors.

**Table 2:** Summary of differentially expressed proteins of each HD stage.

Group Name	Total Proteins Identified (*n* = 1638)	Number of Differentially Over-Expressed Proteins (FC > 1.2, *p* < 0.05)	Number of Differentially Under-Expressed Proteins (FC < −1/1.2, *p* < 0.05)
Asymptomatic HD vs. Control	583	45	35
Early Symptomatic HD vs. Control	534	25	38
Symptomatic Advanced HD vs. Control	521	14	45

**Table 4:** Biological functions and roles of differentially expressed proteins in HD and other neurodegenerative diseases.

Protein Name	UniProt ID	Biological Function	Previously Reported in HD or Neurodegeneration	Ref.
Complement Component 5 (C5)	P01031	Phagocytosis, innate immune response, inflammation	AD, PD ALS, HD	[94]
Complement Component (C7)	Q8TCS7	Membrane attack complex, innate immune response	AD, ALS, HD	[95]
Complement Component (C9)	A0A8Q3SI37	Membrane attack complex, innate and adaptive immune response	AD, HD	[94]
Mannan-Binding Lectin Serine Protease 1 (MASP1)	P48740	Lectin pathway involvement in complement system, activation of MASP2 and MASP3	AD, HD	[94]
Alpha-1-Microglobulin/Bikunin Precursor (AMBP)	P02760	Antioxidant, tissue repair, reed cell homeostasis	AD, HD	[90]
Inter-α Trypsin Inhibitor Heavy Chain 4 (ITIH4)	Q14624	Anti-inflammatory, inflammation and host defense	AD, HD	[93]

## Data Availability

The datasets supporting the conclusions of this article are available in the ProteomeXchange repository [PXD053477 in https://www.ebi.ac.uk/pride/archive/projects/PXD053477/private, accessed on 24 June 2024]. Publicly available datasets used in this study: HD-associated genes (C002017) from DisGeNet database v7.0 (https://www.disgenet.org/, accessed on: 9 May 2024) [46]; HD SNPs from GWAS (https://www.ebi.ac.uk/gwas/, accessed on 9 May 2024) [47]. The codes used for analysis in this study are available upon request from the corresponding authors.

## Supplementary Materials

The following supporting information can be downloaded at https://www.mdpi.com/article/10.3390/cells14151195/s1: Table S1: Demographic and anthropometric characteristics of Cypriot cases and controls; Table S2: All proteins identified from LC-MS/MS analysis across HD stages; Table S3: Differentially Expressed Proteins (DEPs) for asymptomatic HD stage vs. controls; Table S4: Differentially Expressed Proteins (DEPs) for early symptomatic HD stage vs. controls; Table S5: Differentially Expressed Proteins (DEPs) for symptomatic HD stage vs. controls; Figure S1: Venn diagram of all proteins, overlapping across and exclusive to HD stages; Figure S2: Venn diagram of over-expressed and under-expressed proteins across HD stages; Table S6: Gene Ontology (GO) terms for asymptomatic HD vs. controls; Table S7: Gene Ontology (GO) terms for early symptomatic HD vs. controls; Table S8: Gene Ontology (GO) terms for symptomatic advanced HD vs. controls; Table S9: Biomarker filter analysis of protein biomarkers across HD stages; Table S10: Exclusive potential protein biomarkers identified through biomarkers filter analysis; Table S11: Gene Ontology (GO) Terms for Symptomatic Advanced HD vs. Controls; Table S12: Biomarker Filter Analysis of Protein Biomarkers Across HD Stages; Table S13: Exclusive Potential Protein Biomarkers identified through Biomarker Filter Analysis.

## Abbreviations

24-OHC	24S-Hydroxycholesterol
AD	Alzheimer’s Disease
ALS	Amyotrophic Lateral Sclerosis
AGC	Automatic Gain Control
AOO	Age of Onset
AUC	Area Under the Curve
BMI	Body Mass Index
CAG	Cytosine–Adenine–Guanine
CNS	Central Nervous System
CSF	Cerebrospinal Fluid
DDA	Data-Dependent Acquisition
DEP	Differentially Expressed Proteins
DTT	DL-dithiothreitol
ELISA	Enzyme-Linked Immunosorbent Assay
FC	Fold Change
FDR	False Discovery Rate
GO	Gene Ontology
GWAS	Genome-Wide Association Studies
HD	Huntington’s Disease
HTT	Huntingtin
IAA	Iodoacetamide
IPA	Ingenuity Pathway Analysis
iPSCs	Induced Pluripotent Stem Cells
IS	Independence Scale
LC-MS/MS	Liquid Chromatography Tandem Mass Spectrometry
LMM	Linear Mixed-Effects Model
LXR	Liver X Receptor
MSN	Medium Spiny Neurons
mHTT	Mutant Huntingtin
NCE	Normalized Collision Energy
ND	Neurodegenerative Disease
NS	Nervous System
PD	Parkinson’s Disease
PPI	Protein–Protein Interaction Networks
SSRI	Selective Serotonin Reuptake Inhibitors
TBS	Total Behavioral Score
TFC	Total Functional Capacity
TMS	Total Motor Score
UHDRS	Unified Huntington’s Disease Rating Scale
